# Stagnation of histopathological improvement is a predictor of hepatocellular carcinoma development after hepatitis C virus eradication

**DOI:** 10.1371/journal.pone.0194163

**Published:** 2018-03-13

**Authors:** Hiroyuki Motoyama, Akihiro Tamori, Shoji Kubo, Sawako Uchida-Kobayashi, Shigekazu Takemura, Shogo Tanaka, Satoko Ohfuji, Yuga Teranishi, Ritsuzo Kozuka, Etsushi Kawamura, Atsushi Hagihara, Hiroyasu Morikawa, Masaru Enomoto, Yoshiki Murakami, Norifumi Kawada

**Affiliations:** 1 Department of Hepatology, Graduate School of Medicine, Osaka City University, Osaka, Japan; 2 Department of Hepato-Biliary-Pancreatic Surgery, Graduate School of Medicine, Osaka City University, Osaka, Japan; 3 Department of Public Health, Graduate School of Medicine, Osaka City University, Osaka, Japan; Inserm U0152, UMR 5286, FRANCE

## Abstract

**Background:**

Hepatocellular carcinoma (HCC) develops in some patients who achieve sustained virological response (SVR) against hepatitis C virus (HCV) infection via anti-HCV therapy. To examine the pathogenesis of HCC development after HCV eradication, histopathological changes and clinical markers were evaluated in SVR patients.

**Methods:**

Of 654 SVR patients treated with interferon (IFN)-based therapies, 34 patients who had undergone liver biopsy before initiating IFN therapy and after SVR achievement were enrolled: 11 patients with HCC and 23 patients without HCC (male/female, 9/2 and 8/15, respectively: age, 58 ± 5 and 54 ± 11 years, respectively). We compared the clinical and histopathological factors between the two groups. Immunohistochemistry for Cytoglobin (CYGB) and α smooth muscle actin (α-SMA) was also performed.

**Results:**

At baseline, prior to initiating the IFN-based therapy, there were significant differences between the SVR-non-HCC and SVR-HCC groups in the male gender, HBc antibody positivity, prothrombin activity, and histological inflammatory grade. Histopathological evaluation, using the new Inuyama classification system, revealed an improvement in the inflammatory grade, from 2.1 ± 0.6 to 1.0 ± 0.6 (p < 0.0001), whereas the fibrosis stage remained unchanged, from 2.3 ± 0.9 to 2.0 ± 1.2 (p = 0.2749), during the 97 ± 72-month observation period in the SVR-HCC group. Both the grade and stage scores were significantly improved in the SVR-non-HCC group. The area of collagen deposition, evaluated using Sirius red staining, showed a marked decrease, from 18.6 ± 7.6% to 7.7 ± 4.6%, in the SVR-non-HCC group, with no change in the SVR-HCC group. CYGB- and α-SMA-positive hepatic stellate cells (HSCs), indicative of the HSC activated phenotype, remained in the fibrotic tissue of livers among patients in the SVR-HCC group.

**Conclusion:**

Stagnation of fibrosis regression is associated with a high risk for HCC after SVR. HSC activation may inhibit improvement in fibrosis after SVR and potentially contribute to hepatocarcinogenesis.

## Introduction

Hepatitis C virus (HCV) infection is a critical factor in the occurrence of hepatocellular carcinoma (HCC). HCV infection becomes chronic in 80% of cases, with a 10–20% risk of developing cirrhosis over 20 years [1–3]. Once established, cirrhosis carries a 1–5% annual risk of developing HCC. Thus, it is reasonable to speculate that the eradication of HCV prevents HCC development in HCV-infected patients. Previous studies have confirmed that interferon (IFN)-based anti-viral therapy reduces the incidence of HCC, particularly in patients who achieve sustained viral response (SVR) [4–6]. Since recent standard regimens using direct-acting antivirals (DAAs) lead to SVR in more than 95% of HCV-infected non-cirrhotic and compensated cirrhotic patients with HCV genotypes 1 and 2 [7–9], the number of SVR patients worldwide is expected to increase markedly in the future.

However, it has been reported that HCC develops in 2.5–4.2% of SVR patients who previously treated IFN-based therapies [10–14]. Some Japanese studies have reported that old age, high α-fetoprotein (AFP) levels, advanced liver fibrosis at baseline, and past or occult HBV infection to be risk factors for SVR-HCC [15–17]. In the United States, El-Serarg et al. [18] reported older age, the presence of liver cirrhosis at the time of SVR, diabetes, and alcohol abuse to be associated with a higher risk of SVR-HCC. Makiyama et al. [19] speculated that subclinical HCC exists in fibrotic livers prior to the eradication of HCV via IFN therapy. In addition, there are several case reports of HCC being detected more than 10 years after SVR [20–22]. Currently, the guidelines of the American Association for the Study of Liver Diseases (AASLD) recommend follow-up visits after SVR, particularly for patients with advanced fibrosis. However, comprehensive, but cost-effective, criteria are necessary to identify all patients who are at risk for SVR-HCC and, therefore, should be followed after achieving SVR [23].

Hepatic stellate cells (HSCs) are localized in the space of Disse, where they comprise the hepatic sinusoidal vascular wall and function as the main vitamin A storage sites in the liver. Upon liver injury, HSCs undergo activation and trans-differentiate into myofibroblasts that produce collagen and other extracellular matrix (ECM) components, with an increased expression of α smooth muscle actin (α-SMA). Importantly, activated HSCs also produce tissue inhibitor of metalloproteinase 1 (TIMP-1), which inhibits the activity of matrix metalloproteinases along with ECM degradation, leading to ECM accumulation in chronically inflamed livers. Moreover, TIMP-1 and ECM components, particularly Type I collagen, sustain the survival of activated HSCs, thereby enlarging the fibrogenic cell population [24].

We previously showed that Cytoglobin (CYGB) is an excellent marker of human HSCs [25]. CYGB was originally detected in rat HSCs in 2001, and was the fourth globin identified in mammals [26]. CYGB is present in fibroblastic cells that store vitamin A in the visceral organs, including the liver and pancreas. CYGB is thought to facilitate oxygen (O_2_) diffusion through tissues, scavenge nitric oxide and other reactive oxygen species, have a protective function during oxidative stress, and suppress tumorigenesis [27]. Since CYGB is absent in portal myofibroblasts, it is a useful indicator for distinguishing activated human HSCs from portal myofibroblasts, both of which are positive for α-SMA. However, CYGB expression in SVR liver tissue has not yet been studied. Accordingly, the present study was conducted to elucidate the clinical and histopathological characteristics of patients who developed HCC after achieving SVR via IFN-based anti-viral therapy.

## Materials and methods

### Patient population

This was an observational cohort study. At our hospital, 1864 HCV-infected patients were treated with IFN-based anti-viral therapy between 1992 to 2014: 953 males and 911 females; mean age; 54 years (range, 22–74 years). The first liver biopsy was performed before anti-viral therapy. Patients who achieved SVR (n = 654) were repeatedly followed up with laboratory tests and ultrasonography or enhanced computed tomography imaging, every 6 or 12 months, for more than 20 months. Among the SVR patients, 23 patients who did not develop HCC agreed to undergo a second liver biopsy at more than 3 years after the end of IFN-based therapy (SVR-non-HCC). In addition, 11 of the SVR patients developed HCC and were treated surgically (SVR-HCC). We enrolled these 34 patients, whose liver tissue samples, clinical records and reserved serum samples, obtained before and after IFN-based anti-HCV therapy, were available (Table 1). None of the patients showed hepatitis B surface antigen or any diagnostic markers for primary biliary cholangitis, autoimmune liver disease or hemochromatosis.

**Table 1:** Clinical features of the patients at Pre-IFN treatment.

	Non-HCC group (n = 23)	HCC group (n = 11)	*P-value*
Epidemiology			
Sex (male, n (%))	8 (34)	9 (81)	0.01*
Age (year)	54 ± 11	58 ± 5	0.15
HCV genotype, n (%)			
1	14 (60)	8 (72)	
2	9 (40)	3 (28)	
HBc antibody, n (%)	6 (26)	10 (91)	< 0.01*
Diabetes, n (%)	4 (17)	3 (27)	0.5
Alcohol, n (%)	7 (30)	2 (18)	0.39
Body mass index	22.4 ± 3.1	23.1 ± 3.9	0.65
Laboratory data			
AST (IU/L)	30 ± 54	70 ± 43	0.29
ALT (IU/L)	83 ± 60	95 ± 105	0.72
Platelets (x 10^4^/ml)	16.4 ± 5.5	14.6 ± 5.0	0.47
Total bilirubin (mg/dl)	0.8 ± 0.2	0.9 ± 0.3	0.8
PT activity (%)	93 ± 13	79 ± 14	0.04*
Albumin (g/dl)	3.8 ± 0.2	3.8 ± 0.4	0.58
α-fetoprotein (ng/ml)	6.6 ± 7.4	11.9 ± 8.4	0.18
Histopathology			
Stage (F1/2/3/4)	12/6/2/3	1/6/2/2	0.18
Grade (A0/1/2/3)	0/14/6/3	0/2/6/3	0.03*
Treatment, n (%)			
IFN	4 (17)	8 (72)	
IFN/RBV	7 (30)	1 (9)	
PEG-IFN	0 (0)	1 (9)	
PEG-IFN/RBV	12 (53)	1 (9)	
Observation period (months)	86 (38–199)	97 (20–240)	0.64

The degree of liver fibrosis was assessed based on the new Inuyama classification, using the following definitions: F0, no fibrosis; F1, expansion of the portal tracts without linkage; F2, portal expansion with portal-to-portal linkage; F3, extensive portal-to-portal and focal-to-central portal linkage; and F4, cirrhosis. Mean ± SD, median (interquartile range). AST: aspartate aminotransferase, ALT: alanine aminotransferase, PT activity: prothrombin activity, IFN: interferon, RBV: ribavirin, PEG: pegylated.

*: statistical significance.

### Histopathological examination

The obtained liver tissue samples were fixed in 10% formaldehyde, stained with hematoxylin and eosin, and pathologically evaluated for inflammatory activity and fibrosis, according to the new Inuyama classification [28], by one pathologist and one hepatologist. Inflammatory grade was defined as follows: inflammatory grade 0, no or minimal necro-inflammatory change; grade 1, mild necro-inflammatory change; grade 2, moderate necro-inflammatory change; and grade 3, marked necro-inflammatory change, including confluent necrosis, such as zonal and bridging necrosis. The stage of fibrosis was defined as follows: stage 0, no fibrosis; stage 1, expansion of the portal tracts without linkage; stage 2, portal expansion with portal-to-portal linkage; stage 3, extensive portal-to-portal and focal-to-central portal linkage; and stage 4, cirrhosis.

### Immunostaining of human liver tissue samples

For immunohistochemistry, paraffin-embedded sections were dewaxed in xylene and rehydrated in decreasing concentrations of ethanol (xylene: 3 × 3 min; 100% ethanol: 2 × 3 min; 95% ethanol: 3 min; and 70% ethanol: 3 min). All antibodies were diluted in phosphate-buffered saline containing Tween^®^ 20 (PBS-T). Anti-CYGB polyclonal antibody (produced in our laboratory, 1:100) and anti-α-SMA antibody (Dako, Glostrup, Denmark, clone 1A4, 1:100) were utilized to detect HSCs and myofibroblasts, respectively [25]. In brief, the deparaffinized sections were treated with 3% H_2_O_2_ in 100% methanol for 10 min, at room temperature, to block endogenous peroxidase activity. The sections were then pre-incubated in a serum-free protein blocking solution (Dako, Glostrup, Denmark) for 1 hour at room temperature, and subsequently incubated, overnight, with the primary antibodies in a 1:100 dilution, at a temperature of 4°C. Negative controls, with no primary antibody, were used to assess non-specific staining. After being washed, the samples were incubated with horseradish peroxidase-conjugated goat anti-rabbit IgG (Dako, Glostrup, Denmark, 1:200) and rabbit anti-mouse IgG (Dako, Glostrup, Denmark, 1:200) secondary antibodies for 10 min, at room temperature. After being washed again, the samples were reacted with the Liquid DAB+ Substrate Chromogen System (Dako, Glostrup, Denmark). The stained sections were analyzed using a BZ-X710 microscope (Keyence, Osaka, Japan). The sections were also stained with 0.1% (w/v) Sirius red 2 (Direct Red 80; Sigma-Aldrich, Milwaukee, WI, USA), in a saturated aqueous picric acid solution, for 1 hour at room temperature, to visualize collagen fibers. After staining, sections were washed twice, in two different 0.01 N HCl solutions, and mounted using NEW MX reagent (Matsunami Glass Industries, Osaka, Japan).

### Immunofluorescence staining of human liver tissue samples

The paraffin sections were dewaxed and then incubated with a mixture of antibodies against CYGB and α-SMA, as previously described [25]. After rinsing in a PBS, the sections were incubated with a mixture of fluorochrome-conjugated Alexa Fluor 488 goat anti-rabbit IgG (Molecular Probes, Eugene, OR, USA) and Alexa Fluor 594 goat anti-mouse IgG (Molecular Probes, Eugene, OR, USA) secondary antibodies. Sections were then briefly washed and mounted with ProLong Gold Antifade Reagent (Molecular Probes, Eugene, OR, USA). The resulting sections were evaluated under BZ-X710 microscopy (Keyence, Osaka, Japan).

### Morphometry for hepatic fibrosis

For the morphometric image analysis of hepatic fibrosis, areas of the liver sections which stained positively for Sirius red (red) or α-SMA (brown), were captured separately using a CCD camera connected to a macro digital filing system (BZ-H3XF; Keyence, Osaka, Japan). Images of the whole biopsy section were acquired at a magnification of ×200, digitized and consolidated to create one large image, using the analysis application BZ-H3A (Keyence). Sirius red- or α-SMA-positive areas were measured using the measurement module BZ-H3 M (Keyence) and calculated automatically. The percent (%) area of hepatic fibrosis was calculated as the area stained with the selected color divided by the whole tissue area, at a magnification of ×100.

### Statistical analysis

The results of the statistical analyses are presented in bar graphs, with the mean ± SD. Between-group comparisons were evaluated using Student’s *t*-test for continuous data and Fisher's exact test for categorical data, with a p-value < 0.05 indicating statistical significance.

### Ethical considerations

This study protocol complied with the ethical guidelines of the Declaration of Helsinki (2006 version) and was approved by the Ethics Committee of Osaka City University Graduate School of Medicine (approval no. 1123). Written informed consent was obtained from all enrolled patients.

## Results

### Clinical background at the baseline of IFN-based therapy

A total of 34 patients, infected with HCV genotypes 1 (n = 22) and 2 (n = 12), who had achieved SVR via IFN-based therapy, were included in this study. IFN-based therapy included the following: IFN monotherapy (n = 12); IFN ± ribavirin (RBV) (n = 8); pegylated IFN (n = 1); and pegylated IFN ± RBV (n = 13) for the treatment of the 11 SVR-HCC patients and 23 SVR-non-HCC patients. Relevant demographic and clinical variables for the study group are summarized in Table 1. At baseline, prior to initiating IFN-based therapy, there were significant differences between the SVR-non-HCC and SVR-HCC groups with regard to the proportion of males (p = 0.01), HBc antibody positivity (p < 0.01), prothrombin (PT) activity (p = 0.04), and histological inflammatory grade (p = 0.03). The groups were comparable with regard to the following: age (54 ± 11 vs 58 ± 5 years, respectively), serum levels of aspartate aminotransferase (AST, 30 ± 54 vs 70 ± 43 IU/L, respectively), alanine aminotransferase (ALT, 83 ± 60 vs 95 ± 105 IU/L, respectively), albumin (3.8 ± 0.2 *vs* 3.8 ± 0.4 g/dl, respectively), and AFP (6.6 ± 7.4 vs 11.9 ± 8.4 ng/ml, respectively) and observation period (86 ± 39 vs 97 ± 72 months, respectively). Variables with *p-*values < 0.05 in univariate analyses (male sex, alcohol consumption, and obesity) were entered into a multivariable analysis. Male sex was identified as an independent risk factor for HCC, while alcohol and obesity were not retained as predictive factors.

### Clinical background of patients at the second liver biopsy after SVR

The interval from the end of IFN therapy to the detection of HCC was 20–240 months (mean, approximately 7.8 years) in the SVR-HCC patients, which was similar to the time from IFN therapy to the second biopsy in the SVR-non-HCC patients (38–199 months; mean, approximately 7.2 years). At the second liver biopsy, the following significant differences between the SVR-non-HCC and SVR-HCC groups were identified (Table 2): serum AST level (21 ± 7 vs 30 ± 11 IU/L, respectively, p = 0.01), Type IV collagen level 7S (3.72 ± 0.8 vs 4.56 ± 1.1 ng/ml, respectively, p = 0.05), pathological fibrosis stage (p = 0.02), and inflammatory grade (p = 0.01).

**Table 2:** Clinical features of patients at the second liver biopsy after SVR.

	Non-HCC group (n = 23)	HCC group (n = 11)	*P-value*
Epidemiology			
Age (year)	61 ± 13	66 ± 6	0.13
Laboratory data			
AST (IU/L)	21 ± 7	30 ± 11	0.01*
ALT (IU/L)	19 ± 10	23 ± 11	0.13
Platelets (x 10^4^/ml)	18.9 ± 4.2	17.1 ± 3.3	0.75
Total bilirubin (mg/dl)	0.7 ± 0.2	0.7 ± 0.2	0.7
PT activity (%)	103 ± 15	91 ± 20	0.07
Albumin (g/dl)	4.1 ± 0.3	4.2 ± 0.3	0.4
Cholinesterase (IU/L)	301 ± 71	374 ± 121	0.07
P-III-P (U/ml)	0.49 ± 0.1	0.58 ± 0.1	0.06
Type IV collagen 7S (ng/ml)	3.72 ± 0.8	4.56 ± 1.1	0.05*
Histology			
Stage (F1/2/3/4)	18/2/2/1	3/4/2/2	0.02*
Grade (A0/1/2/3)	19/4/0/0	2/7/2/0	0.01*

P-III-P: procollagen III peptide.

*: statistically significant.

### Comparison of histological findings in the SVR-HCC and SVR-non-HCC groups

**Fig 1:**
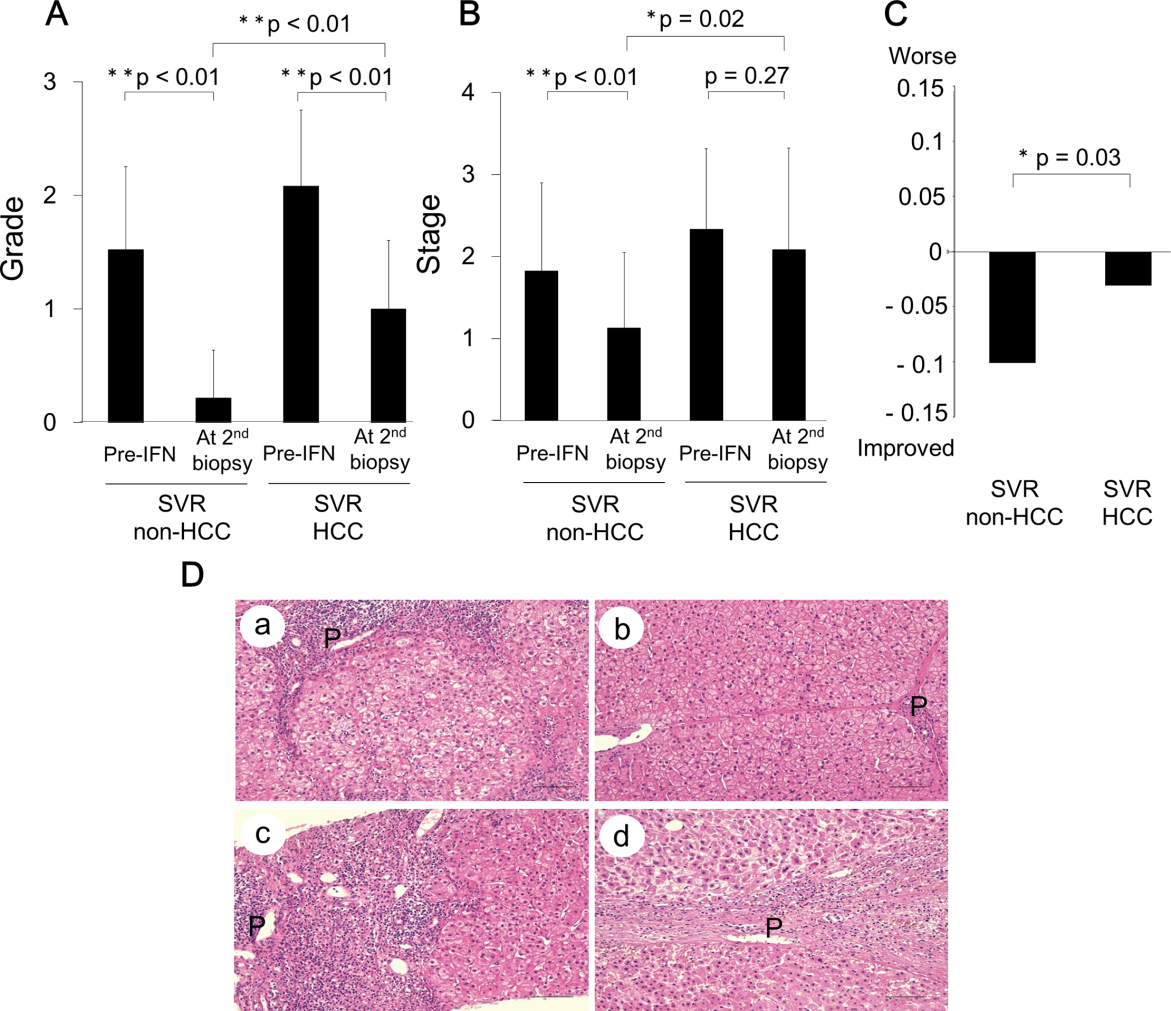
Comparison of inflammation (grade) and fibrosis (stage) scores between SVR patients with and without HCC. (A) Inflammatory grade was significantly improved in SVR patients, with or without HCC. (B) The degree of fibrosis was significantly decreased in the non-HCC group, with negligible change in the HCC group. (C) Changes in the fibrosis score per year, post-IFN treatment, between SVR patients, with or without HCC. (D) Case presentations using hematoxylin and eosin staining. Case 1(a) Pre-IFN treatment liver biopsy from a patient without HCC, showing an obvious nodular organization of hepatocytes, with thick septa. Case 1(b) The second liver biopsy post-IFN treatment, from the same patient, showing a very thin fibrotic septa and negligible inflammation. Case 2(c) Pre-IFN treatment liver biopsy from a patient with HCC, showing nodular fibrosis, with a thick septa and severe inflammation. Case (d) The second liver biopsy obtained at HCC occurrence, from the same patient, showing the presence of a thick fibrotic septa and inflammatory cell accumulation around the portal vein. Bar, 100 μm; P, portal vein; *, p < 0.05; **, p < 0.01.

To evaluate changes in the inflammation grade and fibrosis stage over time, we compared the histopathological factors between the two patient groups, SVR-non-HCC and SVR-HCC. In the SVR-non-HCC group, the grade and stage scores improved from 1.5 ± 0.7 to 0.2 ± 0.4 (p < 0.01) and from 1.8 ± 1 to 1.1 ± 0.9 (p < 0.01), respectively, during the observation period of 86 ± 39 months (Fig 1A and 1B). However, in the SVR-HCC group, the grade and stage scores showed slight differences, from 2.1 ± 0.6 to 1.0 ± 0.6 (p < 0.01) and from 2.3 ± 0.9 to 2.0 ± 1.2 (p = 0.27), respectively. The grade and stage scores were significantly higher in the SVR-HCC than the SVR-non-HCC group (Fig 1A and 1B), even after achieving SVR. When the annual change in the stage score after SVR was calculated, the stage score had improved by 0.101 and 0.031 per year in the SVR-non-HCC and SVR-HCC groups, respectively (Fig 1C, p = 0.03). These results indicate that although SVR improved inflammatory activity in both groups, the degree of residual inflammation was higher and fibrosis regression was reduced in the SVR-HCC group.

Two representative cases from the SVR-HCC and SVR-non-HCC are depicted in Fig 1D. Case 1 is a HCV-infected 42-year-old female who underwent IFN-α monotherapy, from June 2000 to December 2000, at the Osaka City University Medical School Hospital. The patient achieved SVR and did not develop HCC thereafter. Marked regression of liver fibrosis (change from stage 3 to stage 2) and cessation of inflammatory activity (change from grade 2 to 0) were obvious in the liver tissue obtained at the second biopsy, 111 months after SVR (Fig 1D, a and b). In contrast, stagnation of fibrosis regression was observed in Case 2, a HCV-infected 71-year-old male who underwent IFN-α monotherapy from July 2003 to December 2003, at the Osaka City University Medical School Hospital, and developed HCC, 75 months after achieving SVR. In this case, liver fibrosis progressed (from stage 3 to 4), while inflammation improved (from grade 2 to 1; Fig 1D, c and d).

### Collagen deposition and the presence of α-SMA-positive cells in the liver tissue of SVR patients

**Fig 2:**
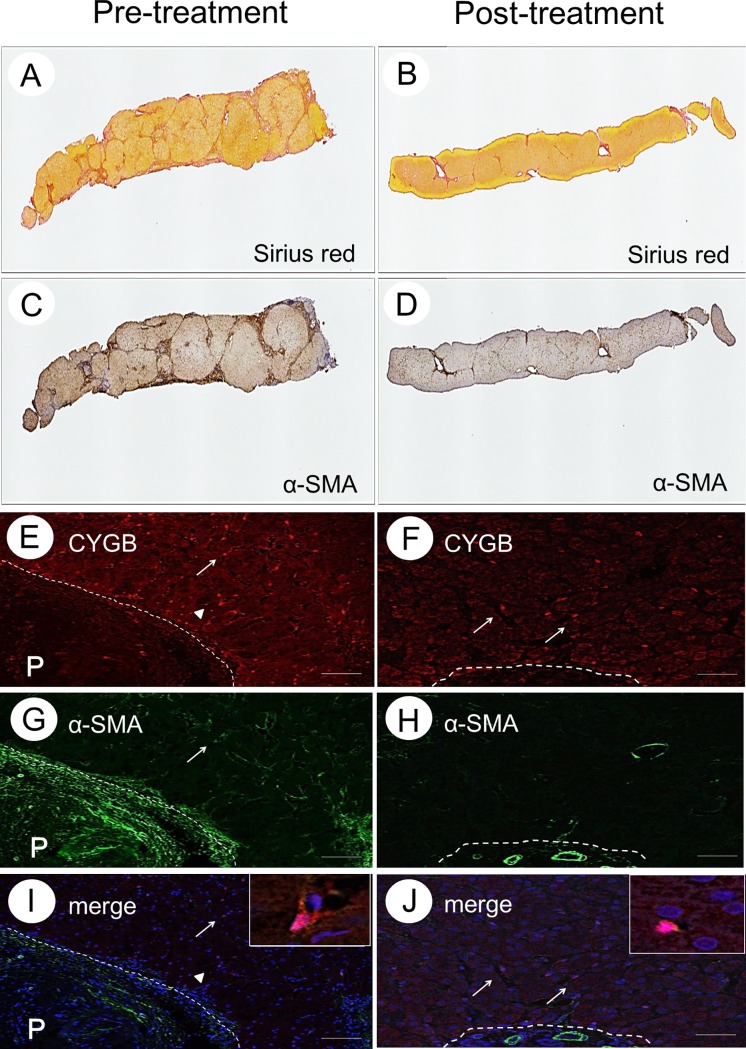
Collagen deposition and the presence of α-SMA-positive cells in liver tissue without HCC. Sirius red staining (A and B), α-SMA immunohistochemistry (C and D), CYGB immunofluorescence staining (E and F), α-SMA immunofluorescence staining (G and H), merged image of CYGB and α-SMA (I and J) of the liver specimen obtained before IFN treatment and after SVR for Case 1. Insets show enlarged views of CYGB/α-SMA-double-positive cells. Bar, 50 μm; P, portal vein.

Collagen deposition and the presence of α-SMA-positive cells were evaluated, semi-quantitatively, using Sirius red staining and immunohistochemistry, respectively, in liver tissue samples obtained before IFN-based therapy and at the second biopsy after achieving SVR. α-SMA is a well-characterized marker of myofibroblasts and activated HSCs in the human liver. Results for a representative case of SVR-non-HCC (Case 1) are shown in Fig 2A and 2B. The calculated area of collagen deposition before IFN therapy was 19.9% of the total tissue area, decreasing to 7.7% at the second biopsy. The calculated α-SMA-positive cell area before IFN therapy was 28.2% of the total tissue area, decreasing to 7.8% at the second biopsy (Fig 2C and 2D). Thus, liver fibrosis markedly regressed in this case.

**Fig 3:**
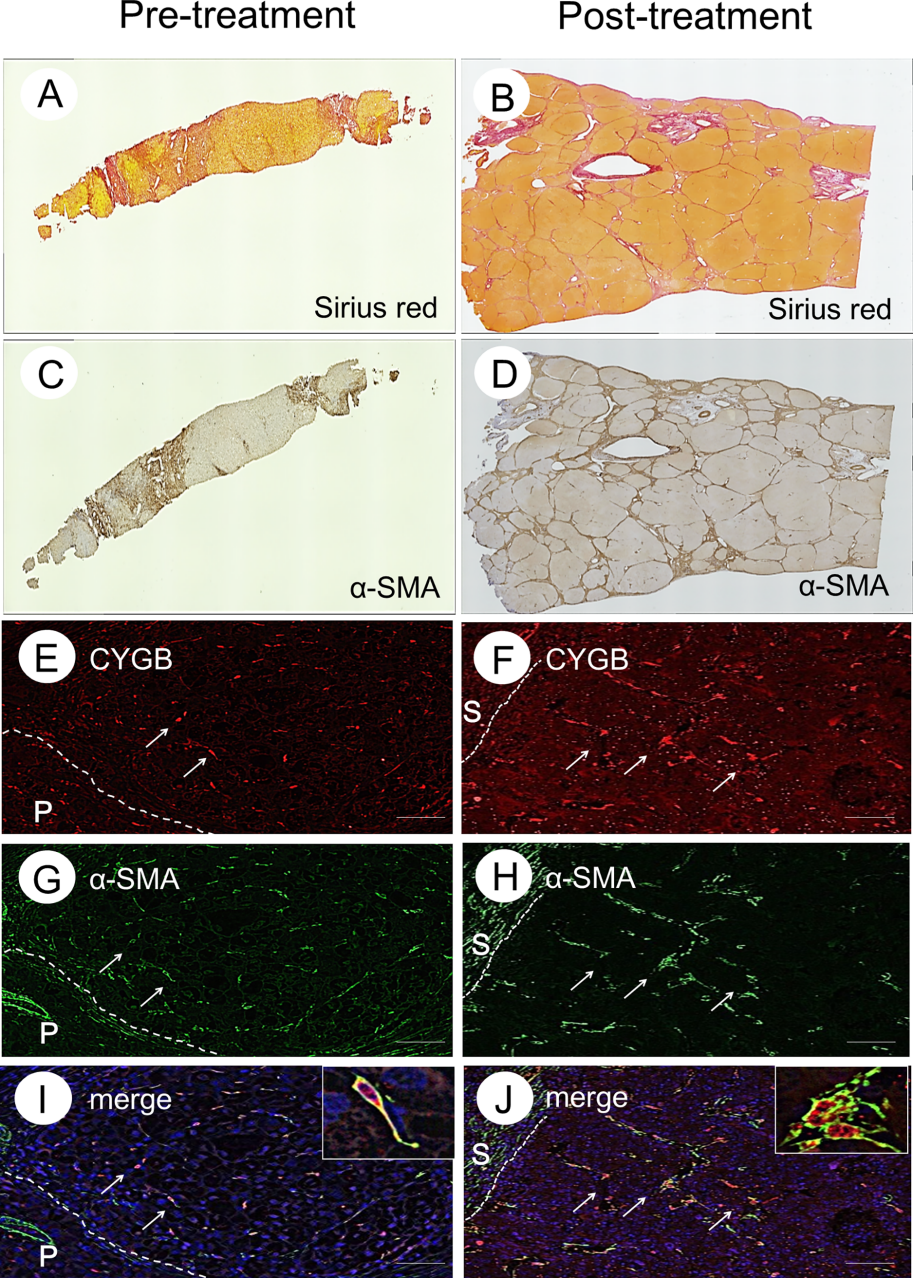
Collagen deposition and the presence of α-SMA-positive cells in liver tissue with HCC. Sirius red staining (A and B), α-SMA immunohistochemistry (C and D), CYGB immunofluorescence staining (E and F), α-SMA immunofluorescence staining (G and H), merged image of CYGB and α-SMA (I and J) before IFN treatment and after SVR from Case 2. Insets show enlarged views of CYGB/α-SMA-double-positive cells. Bar, 50 μm. P, portal vein.

Similar results were obtained for a representative case from the SVR-HCC group (Case 2), shown in Fig 3A–3D. The calculated area of collagen deposition before IFN therapy was 26.4% of the total tissue area, compared to 26.4% at the second biopsy, after SVR (Fig 3A and 3B). The calculated α-SMA-positive cell area before IFN therapy was 24.9% of the total tissue area, increasing to 31.9% at the second biopsy (Fig 3C and 3D). These data reflect the negligible regression in liver fibrosis, even after SVR.

### Morphometric analysis of collagen-deposited and α-SMA-positive cell areas in the liver tissue of SVR patients

**Fig 4:**
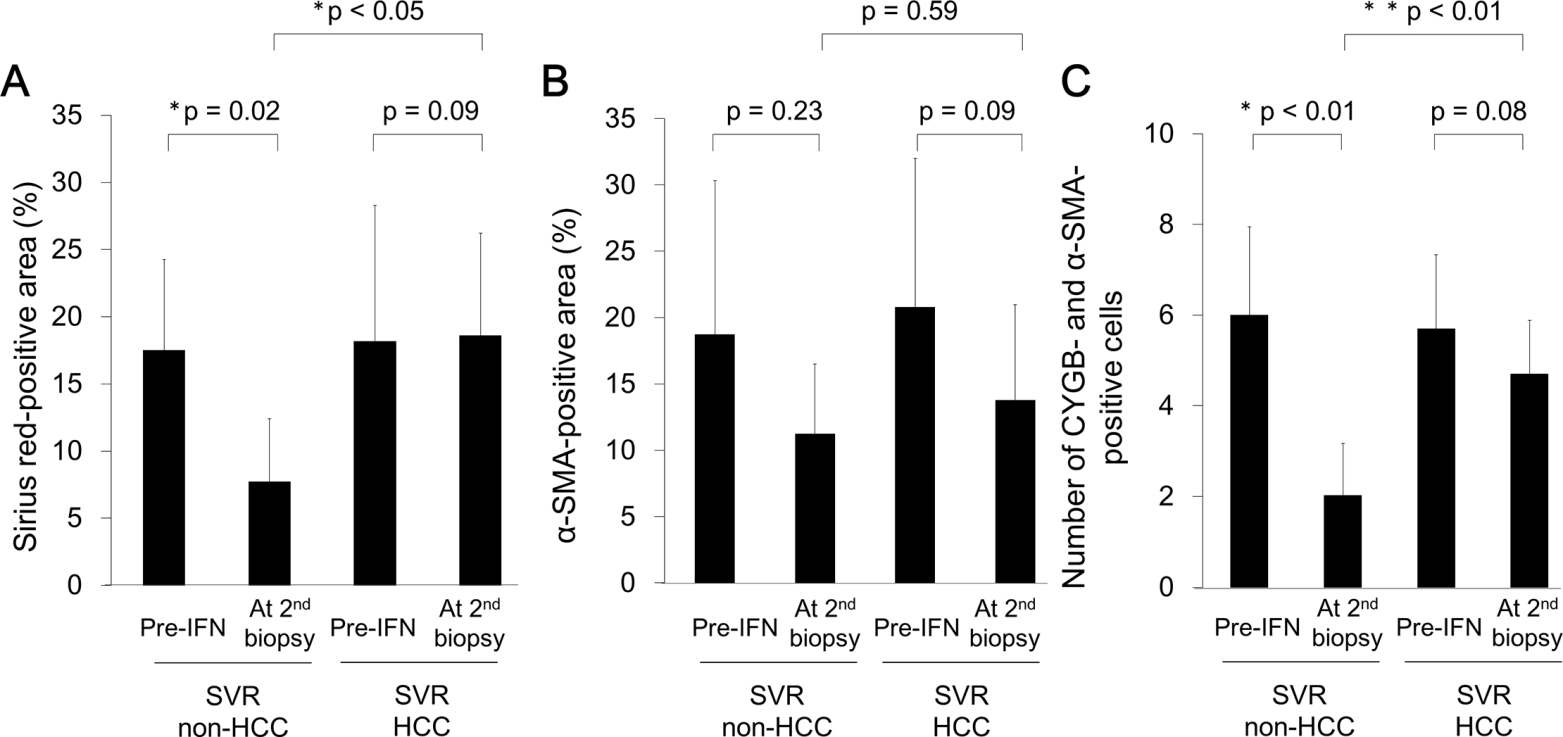
Morphometric analysis. Morphometric analysis of Sirius red-positive (A) and α-SMA-positive (B) areas in SVR patients, with and without HCC. Positive areas for Sirius red and α-SMA immunohistochemistry were determined using the analysis application BZ-H3A (Keyence, Osaka, Japan). Note that both the Sirius red- and α-SMA-positive areas were not improved in the SVR-HCC group. CYGB and α-SMA-positive cells were counted under a × 100 objective. Note that the CYGB and α-SMA-positive cells numbers remained in the SVR-HCC group; * p < 0.05; **, p < 0.01.

Based on the above-mentioned representative results of collagen deposition and α-SMA-positive cell areas in the liver tissue of SVR patients, we evaluated the values obtained from the 11 SVR-HCC patients and 23 SVR-non-HCC patients using morphometry (Fig 4). In the SVR-non-HCC group, the Sirius red-positive area decreased significantly from 17.5 ± 6.7% before IFN-based therapy to 7.7 ± 4.6% at the second biopsy, after SVR (p = 0.02, Fig 4A), with the α-SMA-positive area decreasing from 18.7 ± 11.5% before IFN-based therapy to 11.2 ± 5.2% at the second biopsy, although these differences in α-SMA-positive area were not significant (p = 0.23, Fig 4B). In contrast, in the SVR-HCC group, both the Sirius red-positive and α-SMA-positive areas remained unchanged (Fig 4A and 4B). With regard to the count of CYGB- and α-SMA-positive stellate cells around the fibrotic portal tract, cell counts decreased in the SVR-non-HCC group, from 6 ± 2 before IFN-based therapy to 2 ± 1 at the second biopsy, after SVR (p < 0.01, Fig 4C). In contrast, the cell counts remained unchanged in the SVR-HCC group, from 6 ± 2 before IFN-based therapy to 5 ± 1 at the second biopsy (p = 0.08, Fig 4C).

### Characterization of HSCs and myofibroblasts in the liver tissue of SVR patients

α-SMA was expressed in the cells around the periportal area, and this expression extended along the expansion of collagen deposition, as shown by Sirius red and α-SMA staining in Fig 2A–2D. Activated HSCs and myofibroblasts are known to express α-SMA and to produce collagen in the liver. Using liver tissue samples from patients with chronic hepatitis and cirrhosis caused by HCV infection, we previously demonstrated that CYGB is a unique marker of human HSCs that is negligibly expressed in portal myofibroblasts, which are positive for α-SMA, thy-1, and fibulin 2 [29]. Thus, in the present study, we utilized CYGB as an HSC marker. Here, we describe the representative Case 1. Immunostaining, using a monoclonal antibody against human CYGB, revealed CYGB-positive cells along the sinusoids, throughout the lobule. Hepatocytes and the cells in the portal areas were negative for CYGB (Fig 2E). However, α-SMA-positive cells were observed around the portal vein area and in the vessel walls, with a few α-SMA-positive cells found along the hepatic sinusoids (Fig 2G). CYGB/α-SMA-double-positive cells were present near the fibrotic area in samples obtained before the initiation of IFN-based therapy (Fig 2E, 2G and 2I). However, in the second liver biopsy sample from this patient, the number of α-SMA-positive cells was markedly decreased, with the number of CYGB/α-SMA-double-positive cells being negligible (Fig 2F, 2H and 2J). These observations indicate that activated HSCs, which are positive for both α-SMA and CYGB, disappear when SVR is achieved.

In contrast, Case 2 showed HCC development at 75 months after SVR, and the expression of CYGB and α-SMA in the liver tissue obtained before IFN therapy was nearly identical to that in Case 1. Cells localized along the extended fibrotic septum were showed double-positive for CYGB and α-SMA, suggesting that these cells were activated HSCs in the pretreatment liver biopsy (Fig 3E, 3G and 3I). Notably, the number of CYGB/α-SMA-double-positive cells remained unchanged in the liver tissue, even after achieving SVR (Fig 3F, 3H and 3I). These results indicate that fibrosis regression was impaired and activated HSCs were retained, even in some patients who had achieved SVR, resulting in HCC.

## Discussion

In the study cohort, HCC developed in 20 (3.1%) of the 654 SVR patients over a median follow-up period of 97 months (range, 20–240 months). This rate of HCC is consistent with previously published studies, in which HCC developed in 2.5–4.2% of SVR patients who had undergone IFN-based therapy [10–13]. The present study focused on 23 SVR-non-HCC and 11 SVR-HCC patients in whom pathological diagnosis was evaluated via liver biopsy, before and after SVR. First, comparison of clinical backgrounds between the SVR-non-HCC and SVR-HCC groups showed significant differences with regard to sex and HBc antigen positivity (Table 1). It has already been reported that clinical markers, including these, are risk factors for developing HCC after achieving SVR [30,31]. However, histopathological differences between HCC and non-HCC patients, after SVR, have not been previously reported. The present study clearly demonstrated that hepatic fibrosis general persisted, and even progressed, in patients with HCC after SVR, with regression of fibrosis occurring only rarely. This finding is novel as previous studies have frequently reported regression in fibrosis in liver biopsies obtained from SVR patients during follow-up visits [32,33]. In addition, we showed that stagnation of fibrosis regression was associated with an increase in collagen deposition, as demonstrated using Sirius red staining and successive morphometric analysis of liver tissue samples obtained from SVR-HCC patients, compared to baseline (Figs 3 and 4). However, in all 34 patients, a decrease in inflammatory cell accumulation in the portal area was identified on the second liver biopsy performed after SVR.

The mechanism of impairment in fibrosis regression has not been elucidated in patients with SVR-HCC. The progression and resolution of fibrosis is a complex process involving interactions among parenchymal and non-parenchymal cells. In general, hepatocyte death leads to the release of cellular contents and reactive oxygen species that activate liver-resident macrophages and Kupffer cells to release pro-inflammatory factors, such as tumor necrosis factor α, interleukin (IL)-1β and IL-6, and profibrogenic factors, particularly transforming growth factor (TGF)-β. TGF-β further drives the trans-differentiation of HSCs into myofibroblast-like cells, the main source of ECM proteins, leading to hepatic fibrosis. Therefore, we examined HSCs using an immunohistochemical approach for CYGB and α-SMA. Compared to the SVR-non-HCC group, many activated HSCs, which tested double-positive for CYGB/α-SMA, were detected around the fibrotic area in the SVR-HCC group, even in the absence of HCV. The number of activated HSCs decreases via apoptosis during fibrosis regression [34]. However, no reports have clarified factors inhibiting the apoptosis of activated HSCs in SVR patients. We observed significantly high values of serum AST and Type IV collagen 7S in the SVR-HCC group compared to the SVR-non-HCC group (Table 2). These results are indicative of hepatocyte damage and collagen production in activated HSCs and myofibroblasts, which persists even after the clearance of HCV in SVR-HCC patients. However, epigenetic modifications might occur in HSCs, leading to continuous activation of these cells, as previously reported by Fujita et al. [35]. These points need further clarification through additional experiments using human liver tissue samples.

It has been shown that activated HSCs strongly suppress the response of T-cells, both *in vitro* and *in vivo* [36]. A previous study described the inhibitory effect of HSCs on immune responses, via the regulation of T cells in the HCC microenvironment, indicative of the involvement of HSCs in local immunity responses in the liver [37]. Recently, Matsuura et al. [38] reported that a single nucleotide polymorphism in the intron of the Tolloid-like 1 (TLL1) gene, a member of the bone morphogenetic protein 1/Tolloid-like proteinase family, was strongly associated with HCC development in SVR patients. Notably, the relationship between TLL1 expression and HSC activation, within the context of immunological reactions, has been confirmed in both *in vitro* and *in vivo* models. Therefore, continuous HSC activation might be associated with the development of HCC in SVR patients.

The present study has several limitations that should be acknowledged. First, the number of enrolled patients was small. In addition, it is possible that subclinical HCC was present before the initiation of IFN-based therapy in HCC patients. However, a previous study, using the calculated doubling time of tumor size, reported that the presence of subclinical HCC is unlikely in patients who have not developed HCC within 3 years of the end of IFN-based therapy [39]. In the present study, more than 40 months had passed since the end of IFN-based therapy in 9 of the 11 SVR-HCC patients, indicating that subclinical HCC before treatment initiation, was not likely. Moreover, we did not identify the factors associated with HSC activation after HCV eradication. Previous studies have speculated that diabetes mellitus, obesity, alcohol consumption, and genetic factors are risk factors for HCC in SVR patients [40–43]. Thus, it will be necessary to evaluate the relationship between such factors and the continuous activation of HSCs.

## Conclusions

We showed that stagnation of fibrosis improvement is a risk factor for HCC after SVR. Activated HSCs persisting in the liver tissue of SVR patients may inhibit fibrosis improvement and potentially contribute to hepatocarcinogenesis. The use of IFN-free DAAs is the standard anti-HCV therapy, achieving a high SVR rate in patients with no liver cirrhosis or compensated cirrhosis. However, there is controversy regarding the association between DAAs and HCC, with one study reporting the unexpected development of HCC within one year from the end of DAA use [20], while another study reported a reduced the risk of HCC with DAA use [44, 45]. Thus, further research is needed to evaluate if DAA therapy can improve hepatic fibrosis and prevent the development of HCC, in comparison to the use of IFN-based therapy in SVR patients.
