# Correction: Stagnation of histopathological improvement is a predictor of hepatocellular carcinoma development after hepatitis C virus eradication

**DOI:** 10.1371/journal.pone.0201423

**Published:** 2018-07-24

**Authors:** Hiroyuki Motoyama, Akihiro Tamori, Shoji Kubo, Sawako Uchida-Kobayashi, Shigekazu Takemura, Shogo Tanaka, Satoko Ohfuji, Yuga Teranishi, Ritsuzo Kozuka, Etsushi Kawamura, Atsushi Hagihara, Hiroyasu Morikawa, Masaru Enomoto, Yoshiki Murakami, Norifumi Kawada

In the Funding section, a second grant number from the Japan Society for the Promotion of Science is missing. The correct funding information is as follows: This study was supported in part by the Japan Society for the Promotion of Science grant (JSPS KAKENHI Grant No. JP15K09019, A.T., and JP17H04124, S.O.). Web site; http://www.jsps.go.jp/english/index.html. The funders had no role in study design, data collection and analysis, decision to publish, or preparation of the manuscript.
